# iTRAQ-Based Protein Profiling Provides Insights into the Mechanism of Light-Induced Anthocyanin Biosynthesis in Chrysanthemum (*Chrysanthemum × morifolium*)

**DOI:** 10.3390/genes10121024

**Published:** 2019-12-09

**Authors:** Yan Hong, Mengling Li, Silan Dai

**Affiliations:** 1 School of Landscape Architecture, Beijing Forestry University, No. 35 Tsinghua East Road, Beijing 100083, China; hongy@bjfu.edu.cn (Y.H.); lmling1002@sina.com (M.L.); 2 Beijing Key Laboratory of Ornamental Plants Germplasm Innovation & Molecular Breeding, Beijing 100083, China

**Keywords:** *Chrysanthemum × morifolium*, iTRAQ, low-light stress, proteomics, anthocyanin, photosynthesis

## Abstract

The generation of chrysanthemum (*Chrysanthemum × morifolium*) flower color is mainly attributed to the accumulation of anthocyanins. Light is one of the key environmental factors that affect the anthocyanin biosynthesis, but the deep molecular mechanism remains elusive. In our previous study, a series of light-induced structural and regulatory genes involved in the anthocyanin biosynthetic pathway in the chrysanthemum were identified using RNA sequencing. In the present study, differentially expressed proteins that are in response to light with the capitulum development of the chrysanthemum ‘Purple Reagan’ were further identified using isobaric tags for relative and absolute quantification (iTRAQ) technique, and correlation between the proteomic and the transcriptomic libraries was analyzed. In general, 5106 raw proteins were assembled based on six proteomic libraries (three capitulum developmental stages × two light treatments). As many as 160 proteins were differentially expressed between the light and the dark libraries with 45 upregulated and 115 downregulated proteins in response to shading. Comparative analysis between the pathway enrichment and the gene expression patterns indicated that most of the proteins involved in the anthocyanin biosynthetic pathway were downregulated after shading, which was consistent with the expression patterns of corresponding encoding genes; while five light-harvesting chlorophyll a/b-binding proteins were initially downregulated after shading, and their expressions were enhanced with the capitulum development thereafter. As revealed by correlation analysis between the proteomic and the transcriptomic libraries, GDSL esterase APG might also play an important role in light signal transduction. Finally, a putative mechanism of light-induced anthocyanin biosynthesis in the chrysanthemum was proposed. This study will help us to clearly identify light-induced proteins associated with flower color in the chrysanthemum and to enrich the complex mechanism of anthocyanin biosynthesis for use in cultivar breeding.

## 1. Introduction

Anthocyanins, a class of plant flavonoid metabolites, are almost universal in the flowering plants and provide scarlet to blue colors in flowers, fruits, leaves, and storage organs [1]. The genetic characteristics of the anthocyanin biosynthetic pathway have been well characterized in plant species, such as in model plants Arabidopsis (*Arabidopsis thaliana*) [2], snapdragon (*Antirrhinum majus*) [3], and petunia (*Petunia hybrida*) [4] as well as in horticultural plants *Phalaenopsis* spp. [5] and *Dendrobium* hybrids [6]. Anthocyanin biosynthesis is regulated by not only intracellular signals but also environmental factors [7]. However, it is as yet unclear how the environmental stimuli induce related signals and correspondingly regulate anthocyanin biosynthesis and accumulation in plants.

Light is one of the key environmental factors stimulating anthocyanin biosynthesis and accumulation amongst plants [8]. The ornamental values of flowers and economic values of fruits can be greatly improved by changing their colors through the regulation of light conditions [9]. In general, light exposure increases but shading decreases the concentration of anthocyanins in plants, because under light conditions, specific plant photoreceptors receive light signal and then form a cascade of intracellular second messenger systems by transducing signals to upregulate anthocyanin biosynthetic genes; however, under dark conditions, the whole biological process is suppressed from the very beginning and related genes are downregulated, thereby generating white or light-colored organs [10]. Besides, several transcription factor (TF) genes have also been identified as essential partners of anthocyanin biosynthetic genes in response to light, e.g., *MdMYB1* in apple (*Malus pumila*) [11] as well as *TCP15* [12] and *MYB112* in Arabidopsis [13]. Nevertheless, the deep mechanism of light-induced anthocyanin biosynthesis still remains elusive.

Chrysanthemum (*Chrysanthemum × morifolium*) is a worldwide famous ornamental crop with rich germplasm, whose yield and output values make it a leader in the global flower industry [14]. Compared with other ornamental traits, the phenotypic variation of chrysanthemum flower is particularly rich in colors [15]. Previous studies have shown that only one pathway related to anthocyanin metabolism—the cyanidin metabolic pathway—exists in the chrysanthemum [16,17]. The simple background of pigment metabolism and the light sensitivity of pigment production make the chrysanthemum an ideal model for studies of anthocyanin biosynthesis and the corresponding molecular regulatory mechanism in response to light.

At present, strategies used for functional genomics studies, e.g., gene microarray and RNA sequencing (RNA-Seq), are only based on transcriptional (mRNA) level. It is widely accepted that, however, transcriptional level cannot completely represent post-transcriptional/translational level because correlation of the expressive abundance between genes and proteins is usually weak under the same experimental conditions [18], particularly when the expressive abundance of proteins is very low. Besides, it is almost impossible to judge the complicated post-translational modification, subcellular localization or transfer of proteins, and protein–protein interactions only based on gene expression patterns. Therefore, the reveal of existence form and active patterns of a specific protein relies on the direct study of the protein itself [19], and complementary authentication between different expression levels is essential. Isobaric tags for relative and absolute quantification (iTRAQ) is employed widely with proven value in discovery-based proteomics [20,21], which allows for simultaneous protein identification and quantification obtained at the tandem mass spectrometry (MS/MS) level from peptide fragments and low mass reporter ions, respectively [22]. Since the tags are isobaric, the signal in the mass spectrometer is the sum of the peptide contribution from all samples, so there is a gain in sensitivity [22]. Because of these features, as a complementary method of RNA-Seq, iTRAQ has been widely used for studies on the relationship between environmental factors and plant color changes [23,24,25,26].

The authors previously identified a series of light-induced structural and regulatory genes involved in the anthocyanin biosynthetic pathway in the chrysanthemum using RNA-Seq [27], but failed to answer the questions that whether those key enzymes on this pathway are also in response to light, and whether, how many, and which enzyme(s) involved in other metabolic processes participate in. Here, we further answered these questions by identifying differentially expressed proteins (DEPs) which are in response to light with the capitulum development of the chrysanthemum using iTRAQ. This study will be helpful for improving functional genomic studies in chrysanthemums and will further our understanding of the molecular mechanisms of light-induced anthocyanin biosynthesis and accumulation.

## 2. Materials and Methods

### 2.1. Plant Materials

**Figure 1:**
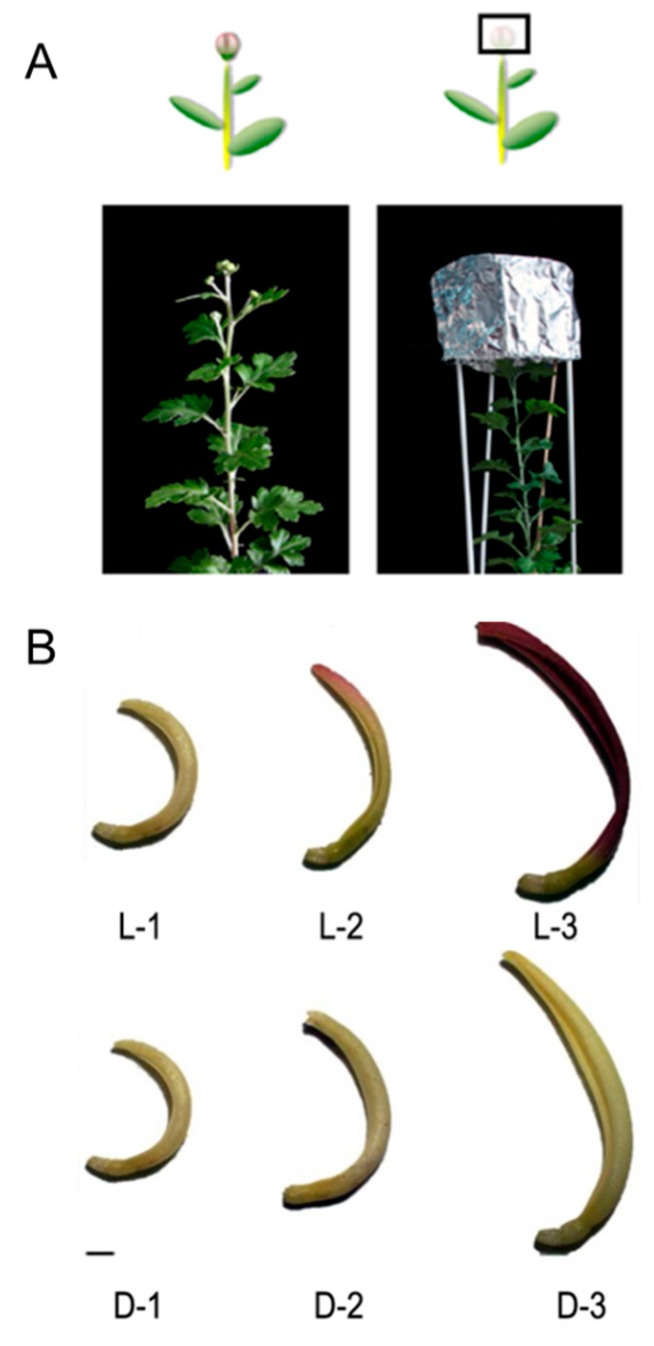
Light and shading treatments used for the chrysanthemum ‘Purple Reagan’ (**A**) and the corresponding color phenotypes of ray florets at each capitulum developmental stage (**B**). L-1, L-2, and L-3 represent samples that were treated under a fluorescent lamp and were sampled at capitulum developmental stages S1, S2, and S3, respectively; D-1, D-2, and D-3 represent samples that were 100% shaded over the whole capitulum using silver papers at each capitulum developmental stage. Scale bar = 0.5 cm. The relative anthocyanin content for each sample was reported by Hong et al. [27].

The capitulum of chrysanthemum cultivar ‘Purple Reagan’ was selected as the experimental material. This cultivar is pentaploid (2*n* = 5x = 45), with the floral competence of 14-leave stage, and the limited inductive photoperiod of 43 d under short daylight (12 h light/12 h dark). A total of three stages were defined during capitulum development, named S1, S2, and S3. Ray floret samples were collected at each capitulum developmental stage under light (fluorescent lamp) and dark (shading using silver paper over the whole capitulum) conditions in September 2018 from an artificial chamber located at the Beijing Forestry University, Beijing, China (intensity of illumination = 50 ± 5 μmol/m^2^/s, temperature = 20 ± 2 °C, and relative humidity = 60%) (Figure 1A). All samples were frozen rapidly in liquid nitrogen and kept at −80 °C. A portion of the samples was used for protein extraction.

### 2.2. Design of Proteomic Libraries

To obtain a general overview of DEPs in response to light, six libraries (L-1, L-2, L-3, D-1, D-2, and D-3) were designed for iTRAQ analysis. L-1, L-2, and L-3 represent samples that were treated under a fluorescent lamp and were sampled at capitulum developmental stages S1, S2, and S3, respectively; D-1, D-2, and D-3 represent samples that were 100% shaded over the whole capitulum using silver papers at each capitulum developmental stage (Figure 1B).

### 2.3. Protein Sample Extraction and iTRAQ Labeling

The extraction of proteins was according to Omar et al. [28]. Briefly, 1 g of each sample was ground into fine powder in liquid nitrogen before transferring into 700 μL of dissolution buffer containing 500 mmol Tris-HCl at pH = 7.5, 150 mmol NaCl, 1 mmol ethylenediamine tetraacetic acid (EDTA), 0.1% Triton-X-100, and 5 mmol dithioerythritol. The mixture was further mixed with 7× protease inhibitor solution, vibrated for 30 min at 4 °C, and centrifuged for 10 min under 12,000× *g* at 4 °C. Purification and purity test of the extracted proteins were carried out by Beijing Proteome Research Center, Beijing, China. Three independent biological replicates were performed for each sample.

A total of 100 μg of proteins were accurately extracted for each sample, which was then mixed with trypsin (Promega, Madison, WI, USA) according to the ratio of proteins:trypsin = 20:1. The mixture was digested for 4 h at 37 °C, followed by deep digestion for 8 h at 37 °C after adding trypsin again with the same ratio. After trypsin digestion, peptides were eliminated using a vacuum centrifugal pump and were redissolved by 0.5 mol/L tetraethylammonium bromide (TEAB; Applied Biosystems, Foster City, CA, USA). iTRAQ labeling was according to the manufacturer’s instruction.

### 2.4. High-Performance Liquid Chromatography (HPLC) Separation

Samples were separated using a Dionex HPLC system equipped with a P680 HPLC pump, UltiMate 3000 autosampler, Thermostatted Column Compartment-100, and Photodiode Array Detector-100 (Thermo Fisher Scientific Inc., Sunnyvale, CA, USA). The iTRAQ-labeled peptide mixtures were reconstituted with 4 mL of buffer A (10 mmol KH_2_PO_4_, 25% acetonitrile, pH = 3.0) and loaded onto a column containing 5 μmol particles. The peptides were eluted at a flow rate of 1 mL/min with a gradient of buffer A for 10 min, 5–60% buffer B (10 mmol KH_2_PO_4_, 1 mol/L KCl, 25% acetonitrile, pH = 3.0) for 27 min, and 60–100% buffer B for 1 min. The system was maintained at 100% buffer B for 1 min before equilibrating with buffer A for 10 min prior to the next injection. Elution was monitored by measuring the absorbance at 214 nm, and fractions were collected every 1 min. The eluted peptides were pooled into 20 fractions, desalted with a column and vacuum dried.

### 2.5. LC–MS/MS Analysis Based on Q-Exactive

The peptides were dissolved in 0.1% formic acid and 2% acetonitrile, and then centrifuged at 13,500× *g* for 20 min. The LC–MS/MS was carried out using a LC-MS/MS Q-Exactive (Thermo Fisher Scientific Inc.) interfaced with an UltiMate 3000 RSLCnano system. The peptide mixture was loaded onto a PepMap C18 trapping column (100 μm i.d., 10 cm long, 3 μm resin from Michrom Bioresources, Auburn, CA, USA) and then separated on the PepMap C18 RP column (2 μm, 75 μm × 150 mm, 100 A) at a flow rate of 300 nL/min. Peptides were eluted from the HPLC column by the application of a linear gradient from 4% buffer B (0.1% formic acid, 80% acetonitrile) to 50% buffer B for 40 min, followed by ramping up to 90% buffer B in 5 min. The eluted peptides were detected by Q-Exactive and MS data were acquired using a data-dependent top 20 method, dynamically choosing the most abundant precursor ions from the survey scan (350–1800 m/z) for high-energy collisional dissociation (HCD) fragmentation. Determination of the target value was based on automatic gain control (AGC). Survey scans were acquired at a resolution of 70,000 at m/z 200, and resolution for HCD spectra was set to 17,500 at m/z 200. Normalized collision energy was 30 eV and the under-fill ratio, which specifies the minimum percentage of the target value likely to be reached at maximum-fill time, was defined as 0.1%. The instrument was run with the peptide recognition mode enabled.

### 2.6. Data Analysis

Identification of the spectra and the DEPs was performed by ThermoFisher Proteome Discoverer (version 1.3; Thermo Fisher Scientific Inc.) and Mascot (version 2.3; Matrix Science Inc., Boston, MA, USA) based on sample intersections between/among the six proteomic libraries. Normalized spectral abundance factor (NSAF) values were used to represent the expressive abundance of proteins [29]. Significantly upregulated proteins were considered if the ratio of NSAF value > 2.0 at *p* ≤ 0.05 between the light and the shading treatments at a specific capitulum developmental stage; significantly downregulated proteins were considered if the ratio of NSAF value < 0.5.

Gene Ontology (GO) functional enrichment analysis of the identified proteins was conducted using Blast2GO (BioBam Bioinformatics, Cambridge, MA, USA). Hierarchical clustering analysis was conducted using MeV (SourceForge Media LLC., La Jolla, CA, USA). Annotation of proteins was identified using BLASTp (NCBI, Bethesda, MD, USA) before KEGG pathway enrichment analysis by KOBAS (version 3.0; NCBI). Correlation between the proteomic and the transcriptomic libraries was analyzed using Microsoft Excel (version 2016; Microsoft, Redmond, WA, USA) based on proteomic data obtained in the present study and transcriptomic data published by Hong et al. [27].

## 3. Results

### 3.1. Overview of Quantitative Proteomics Analysis

In total, 52,527 unique spectra were identified from the chrysanthemum capitulum using the iTRAQ technique based on the six proteomic libraries, which were matched to 16,310 out of 22,202 unique peptides. Subsequently, 5106 raw proteins were assembled. Quality control of the protein data showed that the protein masses were normally distributed based on their expressive abundance (Supplementary Figure S1), indicating a high quality of the identified proteins in the present study which can be used for further analyses.

### 3.2. Correlation Between the Protein Expression and the Capitulum Development under Different Light Treatments

**Figure 2:**
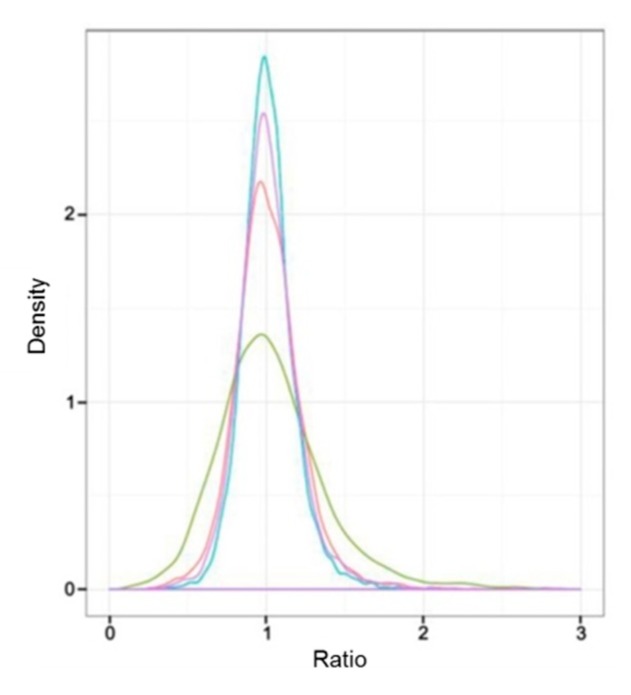
Comparative analysis of the difference of protein expressive abundance between proteomic libraries. X-axis and Y-axis represent the ratio of normalized spectral abundance factor values and the density of the proteins, respectively. Ratio equals to “1” indicates no difference in the expressive abundance. The red, green, blue, and purple curves indicate the expressive abundance of the proteins between proteomic libraries L-1 vs. L-2, L-1 vs. L-3, D-1 vs. D-2, and D-1 vs. D-3, respectively.

Sample intersections between proteomic libraries, including L-1 vs. L-2, L-1 vs. L-3, D-1 vs. D-2, and D-1 vs. D-3, were compared to investigate the correlation between the protein expression and the capitulum development under different light treatments. As a result, the difference of protein expression between L-1 vs. L-3 was the most obvious according to the ratio of NSAF values; L-1 vs. L-2 took the second place. D-1 vs. D-2 and D-1 vs. D-3 did not show obvious differences between each other (Figure 2). These results implied that under the light treatment, the protein expression was more relevant to the capitulum development compared with the shading treatment.

### 3.3. Functional Enrichment of the DEPs

**Figure 3:**
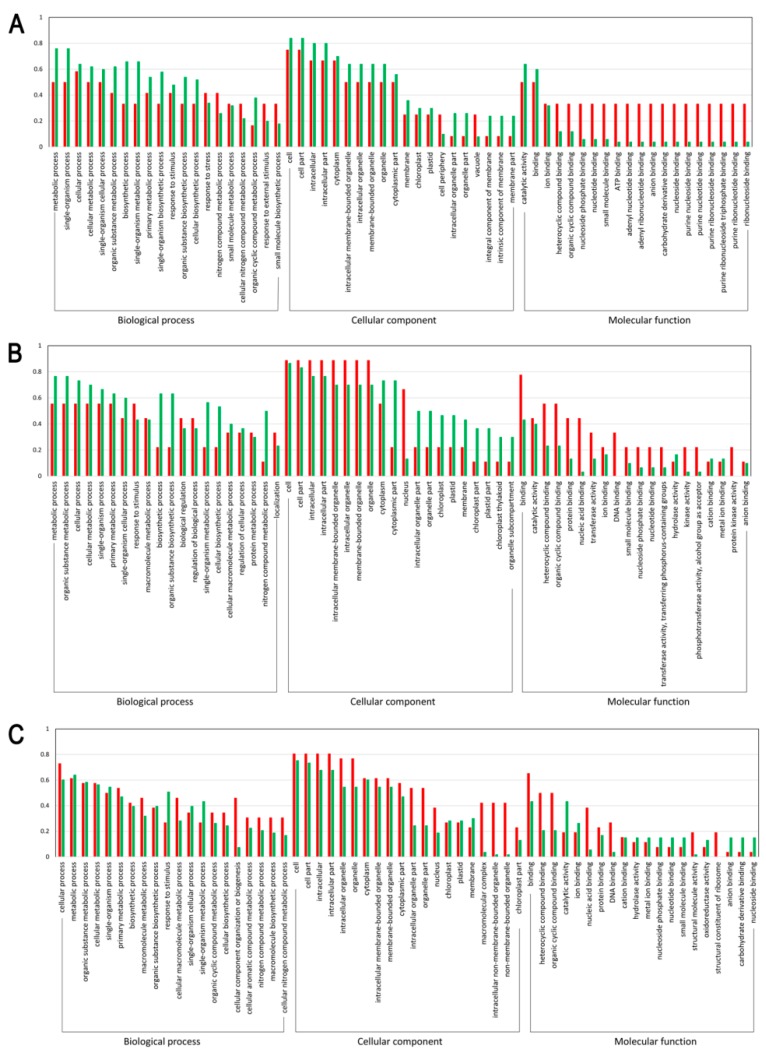
Gene Ontology (GO) functional classification on the differentially expressed proteins (DEPs). (**A**–**C**) indicate DEPs between proteomic libraries L-1 vs. D-1, L-2 vs. D-2, and L-3 vs. D-3, respectively. X-axis and Y-axis represent the annotated GO functional groups and the ratio of normalized spectral abundance factor values, respectively. The red and green bars indicate the up and downregulated proteins, respectively.

To further clarify the functions of the differentially expressed light-responsive proteins, GO functional enrichment analysis was performed. As a result, 55, 32, and 73 DEPs were respectively annotated between the sample intersections of L-1 vs. D-1, L-2 vs. D-2, and L-3 vs. D-3, all of which were categorized into 60 GO functional groups distributed under three main categories: biological processes, cellular components, and molecular functions. Figure 3 clearly illustrated that with the capitulum development from S1 to S3 after shading, (1) the proportion of upregulated proteins related to cellular process, cellular metabolic process, organic substance metabolic process, biosynthetic process, organic substance biosynthetic process, and cellular biosynthetic process under the biological process category increased; (2) under the cellular component category, an increasing trend was found for the proportion of upregulated proteins related to 13 biological processes, such as cell, cell part, intracellular, intracellular part, and cytoplasm; and (3) within the molecular function category, the proportion of downregulated proteins involved in ion binding, nucleoside phosphate binding, nucleotide binding, small molecule binding, anion binding, and nucleoside binding increased (Figure 3). Accordingly, we can conclude that with the capitulum development, the expression of proteins involved in the molecular function category is positively correlated to low light stress, while the opposite is true for those proteins related to biological processes within the biological process and cellular component categories.

**Figure 4:**
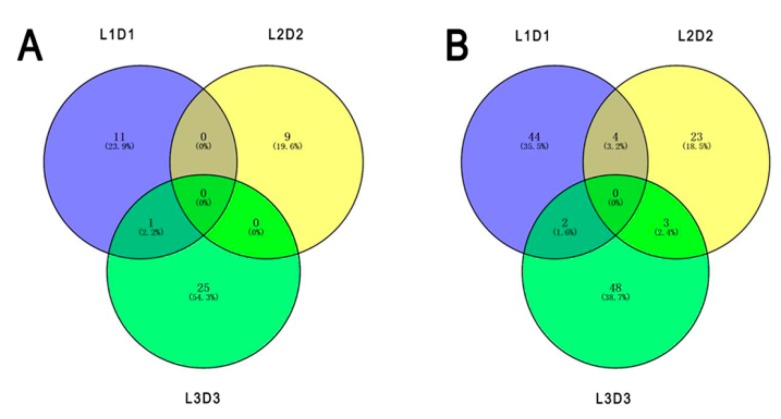
Numbers of specific differentially expressed proteins responding to different light treatments based on the sample intersections among the six proteomic libraries. (**A**) Upregulated proteins. (**B**) Downregulated proteins.

Among the 160 annotated DEPs, 45 and 115 proteins were significantly up- and downregulated after shading, respectively. One unigene (*Unigene28439_All_1*) encoding the upregulated protein and nine unigenes (*CL9080.Contig2_All*, *Unigene6234_All*, *Unigene7244_All*, *Unigene29413_All*, *CL972.Contig2_All*, *CL13575.Contig1_All*, *CL2096.Contig1_All*, *Unigene36275_All*, and *Unigene21072_All*) encoding the downregulated proteins were accurately identified based on the sample intersections among the six proteomic libraries (Figure 4; Table 1).

### 3.4. Clustering and Pathway Enrichment Analyses of the DEPs

**Figure 5:**
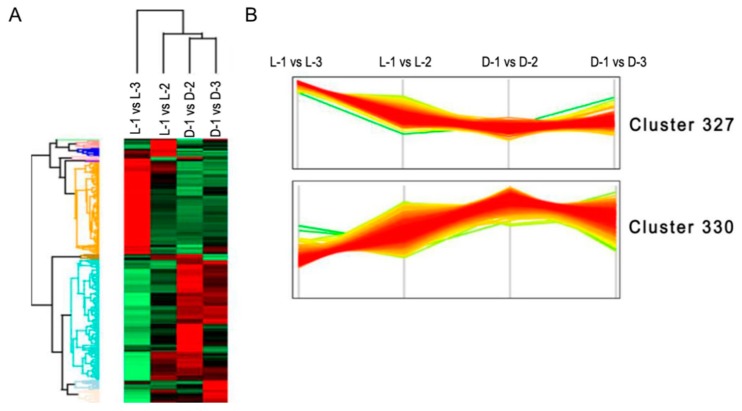
Hierarchical clustering analysis of the differentially expressed proteins (DEPs). (**A**) Undifferentiated clustering analysis of the DEPs between sample intersections of proteomic libraries. The expressive abundance of proteins is indicated using green to red from low to high abundance. (**B**) The expressive pattern of proteins involved in the two primary clusters between sample intersections of proteomic libraries.

Undifferentiated clustering analysis based on the NSAF values of the DEPs showed that most of the DEPs concentrated in two primary clusters: in cluster 327, most of the proteins were obviously downregulated after shading (Figure 5A), while the opposite was true for proteins in cluster 330 (Figure 5B). According to the interpretation of biological characteristics of the enriched items, a majority of the proteins in cluster 327 are involved in anthocyanin biosynthesis (*p* = 0.005), and the proteins in cluster 330 are most likely related to photosynthesis (*p* = 0.001).

**Figure 6:**
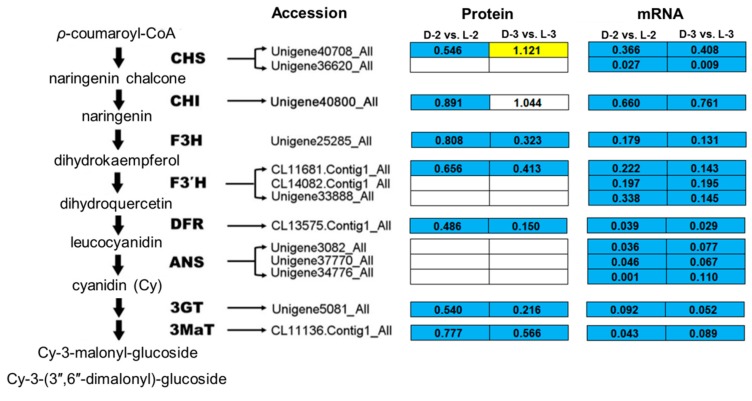
Enrichment analysis of anthocyanin biosynthetic pathway among proteins involved in cluster 327. The yellow, blue, and white squares indicate upregulated, downregulated, and insignificantly changed proteins or genes between sample intersections of libraries, respectively. Protein expressive abundance is indicated by the ratio of normalized spectral abundance factor values, while gene expressive abundance is generated from the transcriptomic data published by Hong et al. [27]. CHS, chalcone synthase; CHI, chalcone isomerase; F3H, flavanone 3-hydroxylase; F3′H, flavonoid-3′-hydroxylase; DFR, dihydroflavonol 4-reductase; ANS, anthocyanin synthase; 3GT, flavonoid 3-*O*-glucosyltransferase; 3MaT, anthocyanin 3-*O*-glucoside-6′′-*O*-malonyltransferase.

**Figure 7:**
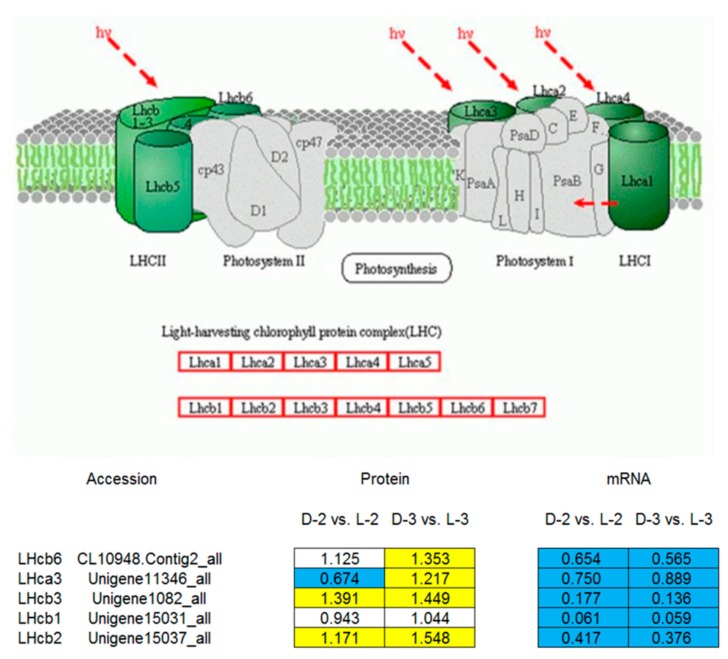
A model of the light-harvesting chlorophyll (LHC) a/b-binding protein complex [30] and pathway enrichment analysis among proteins involved in cluster 330. The yellow, blue, and white squares indicate upregulated, downregulated, and insignificantly changed proteins or genes between sample intersections of libraries, respectively. Protein expressive abundance is indicated by the ratio of normalized spectral abundance factor values, while gene expressive abundance is generated from the transcriptomic data published by Hong et al. [27].

Comparative analysis between the pathway enrichment and the gene expression patterns on mRNA level indicated that, in general, (1) seven out of eight of the proteins involved in the anthocyanin biosynthetic pathway were downregulated after shading except ANS (anthocyanin synthase), which was inconsistent with the gene expression patterns (Figure 6) and (2) five light-harvesting chlorophyll a/b-binding proteins (LHCPs) were initially downregulated after shading, while their expressions increased with the capitulum developed from S1 to S3 (Figure 7).

### 3.5. Correlation between the Proteomic and the Transcriptomic Libraries

**Figure 8:**
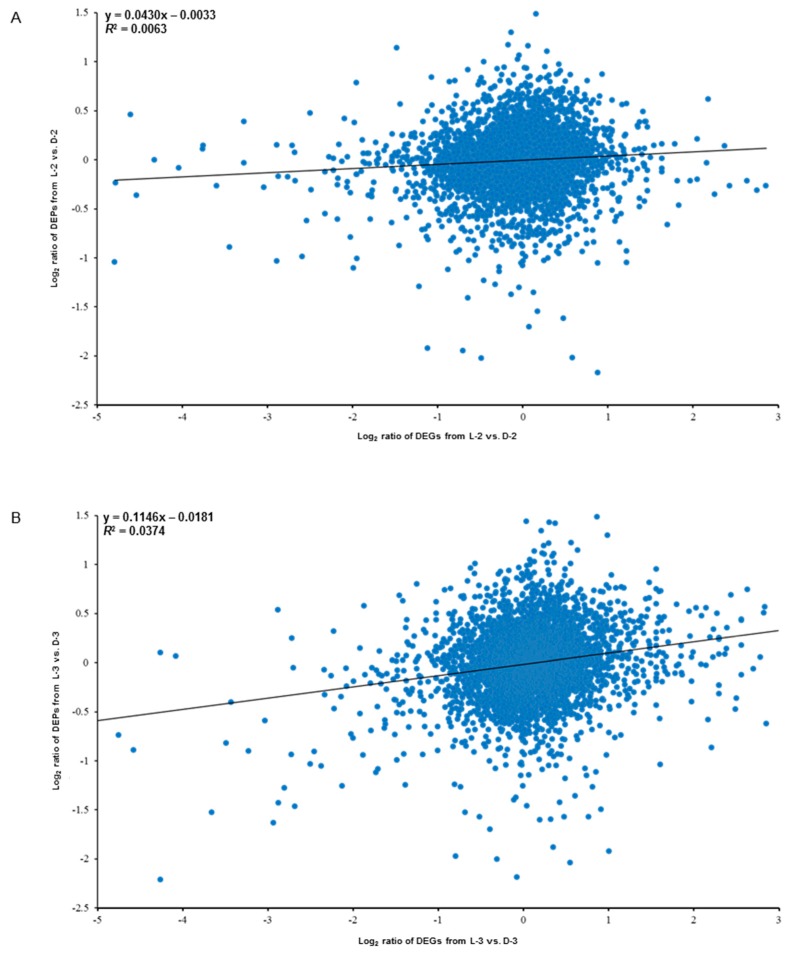
Correlation analysis between the proteomic (data were obtained in the present study) and the transcriptomic (data were published by Hong et al. [27]) libraries between sample intersections of L-2 vs. D-2 (**A**) and L-3 vs. D-3 (**B**). X-axis and Y-axis indicate the conversion of expressive abundance of unigenes/proteins in the transcriptomic/proteomic libraries, respectively. DEGs, differentially expressed genes; DEPs, differentially expressed proteins.

Globally, the expression levels of all the transcripts and their corresponding quantified proteins showed limited correlation: *R*^2^ = 0.0063 for L-2 vs. D-2 (Figure 8A) and *R*^2^ = 0.0374 for L-3 vs. D-3 (Figure 8B). However, (1) the proteins and their encoding genes involved in the anthocyanin biosynthetic pathway were significantly downregulated both on translational and mRNA levels, while plant hormonal proteins and related genes showed an opposite trend and (2) heat shock proteins were significantly downregulated on translational level, which did not show obvious trend on mRNA level (four genes were upregulated and four genes were downregulated). Besides, we found that a GDSL esterase named APG and its encoding gene *CL4884.Contig1_All* were also significantly downregulated both on the two expression levels, indicating this protein might also function in response to light (Table 2).

## 4. Discussion

Light is the key stimulus for anthocyanin biosynthesis [31,32,33]; the positive and negative regulation of anthocyanin pigmentation by light has important physiological implications and application significances for the flower industry [34]. In our previous studies, shading condition inhibits anthocyanin accumulation in the chrysanthemum capitulum based on transcriptional evidence [27,35]. Due to the complex regulatory network of anthocyanin biosynthesis in response to light and the limitations of RNA-Seq technique, direct evidence on translational level must be investigated for complementary authentication. In this proteomic analysis, a number of light-induced proteins involved in different pathways, mainly, anthocyanin biosynthesis and photosynthesis, were identified using iTRAQ, which helped us to clearly identify light-induced proteins associated with flower color in the chrysanthemum and to enrich the complex mechanism of anthocyanin biosynthesis for use in cultivar breeding.

### 4.1. Overview the RNA-Seq and iTRAQ Data

The correlation coefficients of the overall differentially expressed genes (DEGs) [27] and DEP data indicated a positive but very weak correlation between the two light treatments (Figure 8), implying that some post-transcriptional and post-translational modifications or splicing events might occur in the related RNA and protein expression level processes [36], which is consistent with several previous studies [37,38,39,40]. A possible explanation for the poor correlation between transcript levels and protein abundance is that the transcription level can fluctuate more quickly than protein translation and modification processes. Thus, changes in the abundance of a protein occur after the level of its corresponding transcript has stabilized [41]. Our results suggest that iTRAQ is an effective complementary method for profiling candidate proteins mediating specific physiological processes toward RNA-Seq, including the relationship between environmental factors and plant color changes.

### 4.2. Proteins Involved in Anthocyanin Biosynthesis

Anthocyanin biosynthesis is fairly complex and is associated with diverse metabolites including phenylpropanoids and flavonoids [42]. To date, diverse structural genes such as *CHS* (chalcone synthase), *CHI* (chalcone isomerase), *F3H* (flavanone 3-hydroxylase), *F3′H* (flavonoid-3′-hydroxylase), *F3′5′H* (flavonoid-3′,5′-hydroxylase), *DFR* (dihydroflavonol 4-reductase), *ANS*, *3GT* (flavonoid 3-*O*-glucosyltransferase), and *3MaT* (anthocyanin 3-*O*-glucoside-6″-*O*-malonyltransferase) involved in the regulation of anthocyanin biosynthesis have been identified, and their functions in the formation of pigments have been characterized in many plant species [34]. Their protein functions, however, were rarely verified.

A study on petunia demonstrated that anthocyanin biosynthetic enzyme CHS, TF PH4, and signal-transducing protein CONSTANS are key proteins that affect the light-induced pigmentation of petunia flower; a consistent expression pattern between related genes and proteins was found [43]. Most recently, CHI, F3H, and 3GT were found as cor-DEGs-DEPs in eggplant (*Solanum melongena*) in response to light [39], indicating that they might play a broad and important role in the dark to light transition. Similar evidences were also reported for 3GT in grape (*Vitis vinifera*) [44], TF PAP1 (production of anthocyanin pigment 1) and PAP2 in Arabidopsis [45], and TF MYB1 in apple [11]. In the present study, most of the anthocyanin biosynthetic proteins were downregulated after shading except ANS (Figure 6), which is consistent with the reduced transcripts of corresponding genes, the decrease of relative anthocyanin contents, and the fading of ray floret color [27], indicating that reduction in CHS, CHI, F3H, F3’H, DFR, 3GT, and 3MaT after shading immediately results in the fading of chrysanthemum ray florets by repressing anthocyanin biosynthesis. Post-transcriptional modifications and the lag between mRNA appearance and protein synthesis could be plausible explanations for the inconsistency between ANS abundance and the expression of its encoding gene.

The regulatory functions of the other identified enzymes in pathways related to anthocyanin biosynthesis, especially those in the stilbenoid, diarylheptanoid, and gingerol biosynthesis pathways, were not characterized in this study. Combined analyses of metabolomics data may further clarify their functions in relation to the light-induced anthocyanin biosynthesis in the chrysanthemum.

In conclusion, the seven candidate proteins and corresponding encoding genes may be useful for marker-based breeding of new chrysanthemum cultivars under low-light stress.

### 4.3. Proteins Involved in Photosynthesis

LHCP is one of the most abundant proteins of the chloroplast in plants. It roughly accounts for half amount of the chlorophyll involved in photosynthesis. The main function of LHCPs is collecting and transferring light energy to photosynthetic reaction centers [46,47,48]. Many homologous genes encoding LHCPs from various plant species belong to one of the 10 members in the gene family [49]. Four LHCPs of photosystem (PS) I, named LHCI, are encoded by the *Lhca1*, *Lhca2*, *Lhca3*, and *Lhca4* [49]. Three major PS II-associated LHCPs, designated as LHCII and encoded by *Lhcb1*, *Lhcb2*, and *Lhcb3*, are highly homologous and probably form homo- or heterotrimers [49,50]. Three other PS II- associated LHCPs have been designated as minor LHCPs, including inner antenna chlorophyll a-binding complexes CP29, CP26, and CP24 that are encoded by the *Lhcb4*, *Lhcb5*, and *Lhcb6* genes, respectively [50]. The minor LHCPs are monomeric and more closely associated with PS II than the major LHCPs [49,51,52].

Content changes may occur in LHCII complex to improve the tolerance of low-light stress in plants [53]. For example, Laroche et al. [54] found that contents of LHCII complex and chlorophylls significantly increased after transferring *Dunaliella tertiolecta* from high-light to low-light conditions, and the ratio of chlorophyll a/b decreased, indicating that plants may better adapt to low-light stress by increasing the contents of LHCII complex and chlorophyll b in the leaves. In Arabidopsis, LHCII complex content increased when CAO (chlorophyll a oxygenase) overexpressed [55]. Another study by Masuda et al. [56] reported that when external light conditions changed, only *CAO* and *Lhcb* showed consistent expression patterns amongst several chlorophyll synthase genes in *D. tertiolecta*. In the present study, Lhcb1, Lhcb2, Lhcb3, and Lhcb6 were downregulated at the capitulum developmental stage S1 after shading, which was then upregulated with the capitulum developed from S1 to S3 (Figure 7). This finding is consistent with Laroche et al. [54]. Therefore, we speculate that during the adaptive process of low-light stress, intracellular LHCPs in the chrysanthemum capitulum are variably regulated via light signal transduction pathway to increase the LHCII complex content, and accordingly, light energy is more effectively utilized under low-light stress and photoprotection is enhanced. Nevertheless, encoding genes of LHCPs were continuously downregulated after shading with the capitulum development (Figure 7), implying that such mechanism only takes effect on post-transcriptional level.

Besides LHCPs, psaA (PS I), psaH (PS II), and Protease Do-like 8 (PS II) involved in photosynthesis were also identified as DEPs in response to light (Figure 4; Table 1). psaA constitutes not only the core antenna of PS I but also its reaction center [57]. psaH is also an active matter in PS I, but it shows a slower light-response ability compared to the core antenna of PS I, such as psaA and psaB [58]. Protease Do-like 8, a type of chloroplastic protease encoded by *DEGP8*, might involve in chloroplast biosynthesis and PS II repair [59]. All of these proteins were downregulated after shading, suggesting their positive roles in photosynthesis, which is consistent with previous studies [60].

### 4.4. Putative Roles of GDSL Esterase APG

GDSL esterases/lipases are a newly discovered subclass of lipolytic enzymes that are very important and attractive research subjects because of their multifunctional properties, such as broad substrate specificity and regiospecificity [61]. In Arabidopsis and rice (*Oryza sativa*), 108 [62] and 114 [63] members are included in the GDSL esterase family, respectively. GDSL esterases regulate multiple metabolic processes in plants, e.g., plant growth and development such as pollen formation and development [64], root growth [65] and floral development [66] as well as stress tolerance such as water stress [67].

Riemann et al. [68] reported *GER1*, a gene encoding GDSL esterase in rice, involves in the regulation of coleoptile development with the participation of red light and jasmonic acid and is repressed by unknown TF(s) under dark condition. Under red light, GER1 is regulated by PHYA (phytochrome A) and PHYB [69] on post-transcriptional level. In this study, GDSL esterase APG and its encoding gene showed the same expression pattern as anthocyanin biosynthetic genes, i.e., it was downregulated after shading both on transcriptional and translational levels; therefore, we speculate that this protein might also play important role in response to light. Sequence characteristics and promoter structure of the encoding gene (*CL4884.Contig1_All*) and protein–protein interactions need to be further studied in the future to reveal its functions.

## 5. Conclusions

Based on the current knowledge, a putative mechanism of light-induced anthocyanin biosynthesis in the chrysanthemum is proposed. Briefly, in darkness, nuclear-localized COP1 (constitutively photomorphogenic 1) targets positive regulator HY5 (long hypocotyl 5) for ubiquitination and subsequent protein degradation through a 26S proteasome pathway [35]. Negative regulators such as MYB4 and MYB5 [27,70] are active and repress anthocyanin biosynthesis. In light, the activated photoreceptor UVR8 (ultraviolet resistance locus 8) is ubiquitinated by COP1 and targeted for degradation [71]. COP1 is subsequently exported from nucleus allowing nuclear-localized TFs such as HY5, MYB6, and MYB7 [27,35,70] to accumulate and induce expression of structural anthocyanin genes including CHS, CHI, F3H, F3’H, DFR, 3GT, and 3MaT, to generate anthocyanins. This inference, however, needs further verification based on the protein–protein interactions among HY5, anthocyanin biosynthetic genes, and related TFs.

## Figures and Tables

**Table 1:** Functional annotation of the specific differentially expressed proteins responding to different light treatments.

No.	Expressive Pattern of Proteins	Sample Intersection	Encoding Unigenes	Species	Functional Annotation in NCBI-nr	Ko ID
1	Upregulated	L-1, D-1, L-3, D-3	*Unigene28439_All_1*	*Acanthopanax senticosus*	SSR sequence	–
2	Downregulated	L-1, D-1, L-2, D-2	*CL9080.Contig2_All*	*Glycine max*	Protease Do-like 8 (PS II)	K01362
3			*Unigene6234_All*	*Asteraceae* spp.	psaA (PS I)	K02689
4			*Unigene7244_All*	*Vitis vinifera*	Auxin-induced protein 5NG4	–
5			*Unigene29413_All*	*Nicotiana tabacum*	psaH (PS II)	K02695
6		L-2, D-2, L-3, D-3	*CL972.Contig2_All*	*Taraxacum mongolicum*	TO41-1rc mRNA	–
7			*CL13575.Contig1_All*	*Chrysanthemum × morifolium*	DFR	K13082
8			*CL2096.Contig1_All*	*N. tabacum*	Non-specific lipid-transfer protein	–
9		L-1, D-1, L-3, D-3	*Unigene36275_All*	*V. vinifera*	ABC transporter A family member 3	K05643
10			*Unigene21072_All*	*Solanum lycopersicum*	d-galactonate dehydratase	K01684

SSR, Simple sequence repeats; DFR, dihydroflavonol 4-reductase; PS, photosystem.

**Table 2:** Analysis of significant discrete points between the proteomic (data were obtained in the present study) and the transcriptomic (data were published by Hong et al. [27]) libraries among sample intersections of L-2, D-2, L-3, and D-3.

No.	Dispersion	Accession	Species	Functional Annotation in NCBI-nr
1	Log_2_ ratio of DEP < −1 and Log_2_ ratio of DEG < −2	*CL13575.Contig1_All*	*Chrysanthemum × morifolium*	DFR
2		*CL11681.Contig1_All*	*C. × morifolium*	F3’H
3		*Unigene23847_All*	*C. × morifolium*	F5H
4		*Unigene5081_All*	*Lobelia erinus*	3GT
5		*Unigene25285_All*	*Nicotiana tabacum*	F3H
6		*CL4884.Contig1_All*	*Ricinus communis*	APG
7	Log_2_ ratio of DEP > 0.5 and Log_2_ ratio of DEG > 2	*Unigene20184_All*	*Vitis vinifera*	GA3OX4
8		*CL6029.Contig1_All*	*Actinidia deliciosa*	Polygalacturonase inhibitor
9		*Unigene37908_All*	*Elaeis guineensis*	Auxin-repressed protein
10	Log_2_ ratio of DEP < −1.5 and −0.5 < Log_2_ ratio of DEG < 0.5	*CL11963.Contig1_All*	*Helianthus annuus*	17.6 kDa HSP
11		*Unigene37058_All*	*H. annuus*	Nonspecific lipid-transfer protein
12		*CL4402.Contig1_All*	*Lactuca sativa*	HSP90
13		*CL4402.Contig3_All*	*Solanum lycopersicum*	HSP83
14		*Unigene10675_All*	*V. vinifera*	17.4 kDa HSP
15		*CL1186.Contig4_All*	*Ageratina adenophora*	17.7 kDa HSP
16		*Unigene28001_All*	*V. vinifera*	HSP83
17		*Unigene40423_All*	*R. communis*	Putative HSP

DEGs, differentially expressed genes; DEPs, differentially expressed proteins; DFR, dihydroflavonol 4-reductase; F3’H, flavonoid-3’-hydroxylase; F5H, ferulate-5-hydroxylase; 3GT, flavonoid 3-*O*-glucosyltransferase; F3H, flavanone 3-hydroxylase; GA3OX4, gibberellin 3-beta-dioxygenase 4; HSP, heat shock protein.

## Supplementary Materials

The following are available online at https://www.mdpi.com/2073-4425/10/12/1024/s1, Figure S1: Distribution pattern of the raw proteins identified in the present study based on their expressive abundance.

## References

[1] Silva V.O., Freitas A.A., Maçanita A.L., Quina F.H. (2016). Chemistry and photochemistry of natural plant pigments: The anthocyanins. J. Phys. Org. Chem..

[2] Somerville C., Koornneef M. (2002). A fortunate choice: The history of Arabidopsis as a model plant. Nat. Rev. Genet..

[3] Iwashina T. (2015). Contribution to flower colors of flavonoids including anthocyanins: A review. Nat. Prod. Commun..

[4] Zhang H., Koes R., Shang H., Fu Z., Wang L., Dong X., Zhang J., Passeri V., Li Y., Jiang H. (2019). Identification and functional analysis of three new anthocyanin R2R3-MYB genes in Petunia. Plant Direct.

[5] Hsu C.C., Chen Y.Y., Tsai W.C., Chen W.H., Chen H.H. (2015). Three R2R3-MYB transcription factors regulate distinct floral pigmentation patterning in *Phalaenopsis* spp.. Plant Physiol..

[6] Li C., Qiu J., Ding L., Huang M., Huang S., Yang G., Yin J. (2017). Anthocyanin biosynthesis regulation of *DhMYB2* and *DhbHLH1* in *Dendrobium* hybrids petals. Plant Physiol. Biochem..

[7] Tanaka Y., Ohmiya A. (2008). Seeing is believing: Engineering anthocyanin and carotenoid biosynthetic pathways. Curr. Opin. Biotechnol..

[8] Albert N.W., Lewis D.H., Zhang H., Irving L.J., Jameson P.E., Davies K.M. (2009). Light-induced vegetative anthocyanin pigmentation in Petunia. J. Exp. Bot..

[9] Jaakola L., Hohtola A. (2010). Effect of latitude on flavonoid biosynthesis in plants. Plant Cell Environ..

[10] Jaakola L. (2013). New insights into the regulation of anthocyanin biosynthesis in fruits. Trends Plant Sci..

[11] Li Y.Y., Mao K., Zhao C., Zhao X.Y., Zhang H.L., Shu H.R., Hao Y.J. (2012). MdCOP1 ubiquitin E3 ligases interact with MdMYB1 to regulate light-induced anthocyanin biosynthesis and red fruit coloration in apple. Plant Physiol..

[12] Viola I.L., Camoirano A., Gonzalez D.H. (2016). Redox-dependent modulation of anthocyanin biosynthesis by the TCP transcription factor TCP15 during exposure to high light intensity conditions in Arabidopsis. Plant Physiol..

[13] Lotkowska M.E., Tohge T., Fernie A.R., Xue G.P., Balazadeh S., Mueller-Roeber B. (2015). The Arabidopsis transcription factor MYB112 promotes anthocyanin formation during salinity and under high light stress. Plant Physiol..

[14] Teixeira da Silva J.A., Shinoyama H., Aida R., Matsushita Y., Raj S.K., Chen F. (2013). Chrysanthemum biotechnology: Quo vadis?. Crit. Rev. Plant Sci..

[15] Hong Y., Bai X., Sun W., Fu J. (2012). The numerical classification of chrysanthemum flower color phenotype. Acta Hort. Sin..

[16] Nakayama M., Koshioka M., Shibata M., Hiradate S., Sugie H., Yamaguchi M.A. (1997). Identification of cyanidin 3-*O*-(3″,6″-*O*-dimalonyl-β-glucopyranoside) as a flower pigment of Chrysanthemum (*Dendranthema grandiflorum*). Biosci. Biotechnol. Biochem..

[17] Sun W., Li C.H., Wang L.S., Dai S.L. (2010). Analysis of anthocyanins and flavones in different-colored flowers of chrysanthemum. Chin. Bullet. Bot..

[18] Liu Y., Chaturvedi P., Fu J., Cai Q., Weckwerth W., Yang P. (2016). Induction and quantitative proteomic analysis of cell dedifferentiation during callus formation of lotus (*Nelumbo nucifera* Gaertn. spp. *baijianlian*). J. Proteomics.

[19] Wang J., Wang X.R., Zhou Q., Yang J.M., Guo H.X., Yang L.J., Liu W.Q. (2016). iTRAQ protein profile analysis provides integrated insight into mechanisms of tolerance to TMV in tobacco (*Nicotiana tabacum*). J. Proteomics.

[20] Treumann A., Thiede B. (2010). Isobaric protein and peptide quantification: Perspectives and issues. Exp. Rev. Proteomics.

[21] Noirel J., Evans C., Salim M., Mukherjee J., Yen Ow S., Pandhal J., Khoa Pham T., Biggs C.A., Wright P.C. (2011). Methods in quantitative proteomics: Setting iTRAQ on the right track. Curr. Proteomics.

[22] Evans C., Noirel J., Ow S.Y., Salim M., Pereira-Medrano A.G., Couto N., Pandhal J., Smith D., Pham T.K., Karunakaran E. (2012). An insight into iTRAQ: Where do we stand now?. Anal. Bioanal. Chem..

[23] Chu P., Yan G.X., Yang Q., Zhai L.N., Zhang C., Zhang F.Q., Guan R.Z. (2015). iTRAQ-based quantitative proteomics analysis of *Brassica napus* leaves reveals pathways associated with chlorophyll deficiency. J. Proteomics.

[24] Xie H., Yang D.H., Yao H., Bai G., Zhang Y.H., Xiao B.G. (2016). iTRAQ-based quantitative proteomic analysis reveals proteomic changes in leaves of cultivated tobacco (*Nicotiana tabacum*) in response to drought stress. Biochem. Biophys. Res. Commun..

[25] Zhou H., Yu Z., Ye Z. (2018). Key proteins associated to coloured compounds of peach peel using iTRAQ proteomic techniques during development and postharvest. Sci. Hortic..

[26] Zhou H., Yu Z., Ye Z. (2019). Effect of bagging duration on peach fruit peel color and key protein changes based on iTRAQ quantitation. Sci. Hortic..

[27] Hong Y., Tang X., Huang H., Zhang Y., Dai S. (2015). Transcriptomic analyses reveal species-specific light-induced anthocyanin biosynthesis in chrysanthemum. BMC Genomics.

[28] Omar A.A., Song W.Y., Grosser J.W. (2007). Introduction of Xa21, a Xanthomonas-resistance gene from rice, into ‘Hamlin’ sweet orange [*Citrus sinensis* (L.) Osbeck] using protoplast-GFP co-transformation or single plasmid transformation. J. Hortic. Sci. Biotechnol..

[29] Florens L., Carozza M.J., Swanson S.K., Fournier M., Coleman M.K., Workman J.L., Washburn M.P. (2006). Analyzing chromatin remodeling complexes using shotgun proteomics and normalized spectral abundance factors. Methods.

[30] Yang Z., Li W., Su X., Ge P., Zhou Y., Hao Y., Shu H., Gao C., Cheng S., Zhu G. (2019). Early response of radish to heat stress by strand-specific transcriptome and miRNA analysis. Int. J. Mol. Sci..

[31] Takos A.M., Jaffé F.W., Jacob S.R., Bogs J., Robinson S.P., Walker A.R. (2006). Light-induced expression of a MYB gene regulates anthocyanin biosynthesis in red apples. Plant Physiol..

[32] Ampomah-Dwamena C., McGhie T., Wibisono R., Montefiori M., Hellens R.P., Allan A.C. (2009). The kiwifruit lycopene beta-cyclase plays a significant role in carotenoid accumulation in fruit. J. Exp. Bot..

[33] Cazzonelli C.I., Pogson B.J. (2010). Source to sink: Regulation of carotenoid biosynthesis in plants. Trends Plant Sci..

[34] Das P.K., Geul B., Choi S.B., Yoo S.D., Park Y.I. (2011). Photosynthesis-dependent anthocyanin pigmentation in Arabidopsis. Plant Signal. Behav..

[35] Hong Y., Yang L., Li M., Dai S. (2016). Comparative analyses of light-induced anthocyanin accumulation and gene expression between the ray florets and leaves in chrysanthemum. Plant Physiol. Biochem..

[36] Pandey A., Mann M. (2000). Proteomics to study genes and genomes. Nature.

[37] Li J.M., Huang X.S., Li L.T., Zheng D.M., Xue C., Zhang S.L., Wu J. (2015). Proteome analysis of pear reveals key genes associated with fruit development and quality. Planta.

[38] Muers M. (2011). Gene expression: Transcriptome to proteome and back to genome. Nat. Rev. Genet..

[39] Li J., Ren L., Gao Z., Jiang M., Liu Y., Zhou L., He Y., Chen H. (2017). Combined transcriptomic and proteomic analysis constructs a new model for light-induced anthocyanin biosynthesis in eggplant (*Solanum melongena* L.). Plant Cell Environ..

[40] Luo X., Cao D., Li H., Zhao D., Xue H., Niu J., Chen L., Zhang F., Cao S. (2018). Complementary iTRAQ-based proteomic and RNA sequencing-based transcriptomic analyses reveal a complex network regulating pomegranate (*Punica granatum* L.) fruit peel colour. Sci. Rep..

[41] Chen J., Liu S.S., Kohler A., Yan B., Luo H.M., Chen X.M., Guo S.X. (2017). iTRAQ and RNA-Seq analyses provide new insights into regulation mechanism of symbiotic germination of *Dendrobium officinale* seeds (Orchidaceae). J. Proteome Res..

[42] van Tunen A.J., Mur L.A., Recourt K., Gerats A.G., Mol J.N. (1991). Regulation and manipulation of flavonoid gene expression in anthers of petunia: The molecular basis of the Po mutation. Plant Cell.

[43] Berenschot A.S., Quecini V. (2014). A reverse genetics approach identifies novel mutants in light responses and anthocyanin metabolism in petunia. Physiol. Mol. Biol. Plants.

[44] Niu N., Cao Y., Duan W., Wu B., Li S. (2013). Proteomic analysis of grape berry skin responding to sunlight exclusion. J. Plant Physiol..

[45] Maier A., Schrader A., Kokkelink L., Falke C., Welter B., Iniesto E., Rubio V., Uhrig J.F., Hülskamp M., Hoecker U. (2013). Light and the E3 ubiquitin ligase COP1/SPA control the protein stability of the MYB transcription factors PAP 1 and PAP 2 involved in anthocyanin accumulation in Arabidopsis. Plant J..

[46] Bellafiore S., Barneche F., Peltier G., Rochaix J.D. (2005). State transitions and light adaptation require chloroplast thylakoid protein kinase STN7. Nature.

[47] Niyogi K.K., Li X.P., Rosenberg V., Jung H.S. (2004). Is PsbS the site of non-photochemical quenching in photosynthesis?. J. Exp. Bot..

[48] Szabó I., Bergantino E., Giacometti G.M. (2005). Light and oxygenic photosynthesis: Energy dissipation as a protection mechanism against photo-oxidation. EMBO Rep..

[49] Teramoto H., Ono T., Minagawa J. (2001). Identification of *Lhcb* gene family encoding the light-harvesting chlorophyll-a/b proteins of photosystem II in *Chlamydomonas reinhardtii*. Plant Cell Physiol..

[50] Jansson S. (1999). A guide to the *Lhc* genes and their relatives in Arabidopsis. Trends Plant Sci..

[51] Koziol A.G., Borza T., Ishida K., Keeling P., Lee R.W., Durnford D.G. (2007). Tracing the evolution of the light-harvesting antennae in chlorophyll a/b-containing organisms. Plant Physiol..

[52] Dittami S.M., Michel G., Collén J., Boyen C., Tonon T. (2010). Chlorophyll-binding proteins revisited-a multigenic family of light-harvesting and stress proteins from a brown algal perspective. BMC Evol. Biol..

[53] Zhao H., Lou Y., Sun H., Li L., Wang L., Dong L., Gao Z. (2016). Transcriptome and comparative gene expression analysis of *Phyllostachys edulis* in response to high light. BMC Plant Biol..

[54] Laroche J., Mortain-Bertrand A., Falkowski P.G. (1991). Light intensity-induced changes in *cab* mRNA and light harvesting complex II apoprotein levels in the unicellular chlorophyte *Dunaliella tertiolecta*. Plant Physiol..

[55] Tanaka R., Koshino Y., Sawa S., Ishiguro S., Okada K., Tanaka A. (2001). Overexpression of chlorophyllide a oxygenase (*CAO*) enlarges the antenna size of photosystem II in *Arabidopsis thaliana*. Plant J..

[56] Masuda T., Tanaka A., Melis A. (2003). Chlorophyll antenna size adjustments by irradiance in *Dunaliella salina* involve coordinate regulation of chlorophyll a oxygenase (*CAO*) and *Lhcb* gene expression. Plant Mol. Biol..

[57] Kunugi M., Satoh S., Ihara K., Shibata K., Yamagishi Y., Kogame K., Obokata J., Takabayashi A., Tanaka A. (2016). Evolution of green plants accompanied changes in light-harvesting systems. Plant Cell Physiol..

[58] Yamamoto Y.Y., Nakamura M., Kondo Y., Tsuji H., Obokata J. (1995). Early light-response of *psaD*, *psaE* and *psaH* gene families of photosystem I in *Nicotiana sylvestris*: *PSI-D* has an isoform of very quick response. Plant Cell Physiol..

[59] Kley J., Schmidt B., Boyanov B., Stolt-Bergner P.C., Kirk R., Ehrmann M., Knopf R.R., Naveh L., Adam Z., Clausen T. (2011). Structural adaptation of the plant protease *Deg1* to repair photosystem II during light exposure. Nat. Struct. Mol. Biol..

[60] Roose J.L., Frankel L.K., Mummadisetti M.P., Bricker T.M. (2016). The extrinsic proteins of photosystem II: Update. Planta.

[61] Akoh C.C., Lee G.C., Liaw Y.C., Huang T.H., Shaw J.F. (2004). GDSL family of serine esterases/lipases. Prog. Lipid Res..

[62] Ling H. (2008). Sequence analysis of *GDSL* lipase gene family in *Arabidopsis thaliana*. Pak. J. Biol. Sci..

[63] Chepyshko H., Lai C.P., Huang L.M., Liu J.H., Shaw J.F. (2012). Multifunctionality and diversity of *GDSL* esterase/lipase gene family in rice (*Oryza sativa* L. *japonica*) genome: New insights from bioinformatics analysis. BMC Genomics.

[64] Mayfield J.A., Fiebig A., Johnstone S.E., Preuss D. (2001). Gene families from the *Arabidopsis thaliana* pollen coat proteome. Science.

[65] Takahashi K., Shimada T., Kondo M., Tamai A., Mori M., Nishimura M., Hara-Nishimura I. (2009). Ectopic expression of an esterase, which is a candidate for the unidentified plant cutinase, causes cuticular defects in *Arabidopsis thaliana*. Plant Cell Physiol..

[66] Chen C.C., Fu S.F., Lee Y.I., Lin C.Y., Lin W.C., Huang H.J. (2012). Transcriptome analysis of age-related gain of callus-forming capacity in Arabidopsis hypocotyls. Plant Cell Physiol..

[67] Park J.J., Jin P., Yoon J., Yang J.I., Jeong H.J., Ranathunge K., Schreiber L., Franke R., Lee I.J., An G. (2010). Mutation in *Wilted Dwarf and Lethal 1* (*WDL1*) causes abnormal cuticle formation and rapid water loss in rice. Plant Mol. Biol..

[68] Riemann M., Gutjahr C., Korte A., Riemann M., Danger B., Muramatsu T., Bayer U., Waller F., Furuya M., Nick P. (2007). *GER1*, a GDSL motif-encoding gene from rice is a novel early light-and jasmonate-induced gene. Plant Biol..

[69] Takano M., Inagaki N., Xie X., Yuzurihara N., Hihara F., Ishizuka T., Yano M., Nishimura M., Miyao A., Hirochika H. (2005). Distinct and cooperative functions of phytochromes A, B, and C in the control of deetiolation and flowering in rice. Plant Cell.

[70] Hong Y., Li M., Dai S. (2019). Ectopic expression of multiple chrysanthemum (*Chrysanthemum × morifolium*) R2R3-MYB transcription factor genes regulates anthocyanin accumulation in tobacco. Genes.

[71] Yang Y., Yang X., Jang Z., Chen Z., Ruo X., Jin W., Wu Y., Shi X., Xu M. (2018). UV RESISTANCE LOCUS 8 from *Chrysanthemum morifolium* Ramat (CmUVR8) plays important roles in UV-B signal transduction and UV-B-induced accumulation of flavonoids. Front. Plant Sci..

