# Supplementary material for: iTRAQ-Based Protein Profiling Provides Insights into the Mechanism of Light-Induced Anthocyanin Biosynthesis in Chrysanthemum (Chrysanthemum × morifolium)
Source: Genes (Basel). 2019 Dec 9;10(12):1024. doi: 10.3390/genes10121024 (PMC6947405; doi:10.3390/genes10121024)
Supplement: Supplementary file 1 [file genes-10-01024-s001.pdf]

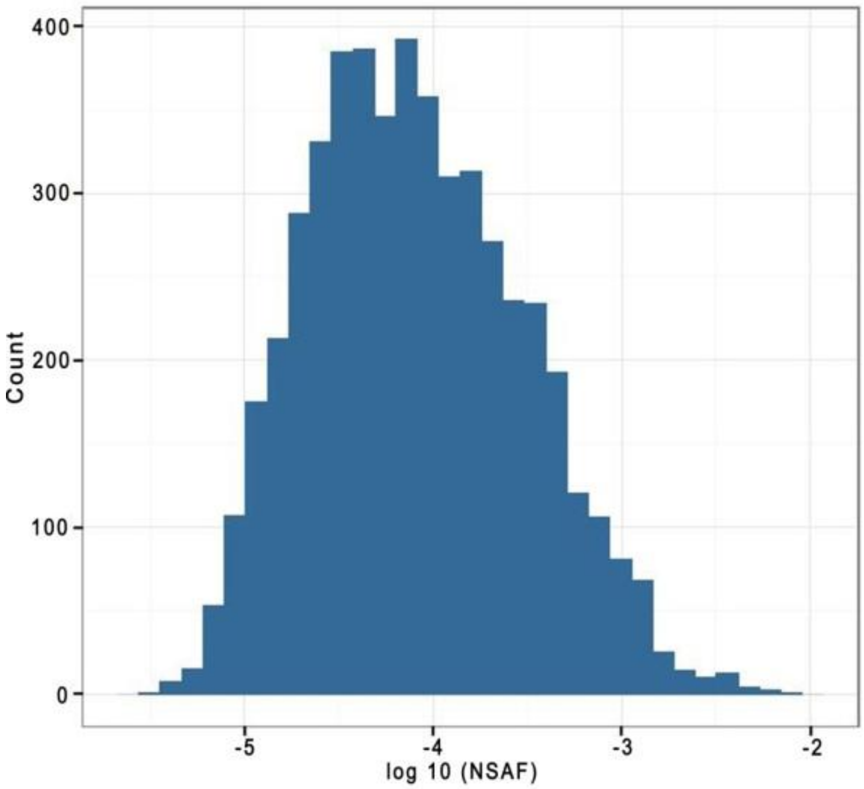

Figure S1. Distribution pattern of the raw proteins identified in the present study based on their expressive abundance. NSAF, normalized spectral abundance factor.
